# Mediated depictions of mental health, chronic care and literacy: a narrative analysis of Randall’s mental health journey in the television series, *This is Us*

**DOI:** 10.3389/fpsyt.2023.1204973

**Published:** 2023-06-16

**Authors:** Kelly E. Tenzek, Tahleen A. Lattimer, Kyle Heneveld, Emily Lapan, Madison Neurohr, Stephanie Gillis

**Affiliations:** Department of Communication, University at Buffalo, The State University of New York, Buffalo, NY, United States

**Keywords:** mental health, literacy, chronic care model (CCM), mediated depictions, narrative, anxiety

## Abstract

**Introduction:**

Mental health and delivery of care is a global issue, that was only magnified by COVID-19. Over the past 3 years, people’s time spent watching television increased, while the way that mental health care was delivered changed. Audiences can gain insight into mental health issues through positive or negative mediated depictions on television. We argue that mental health is a chronic condition and the importance of literacy through different domains is critical for how the characters in media content and audience viewers make sense of mental health.

**Method:**

The current study uses qualitative narrative analysis to examine the narrative probability and fidelity at the intersection of mental health depictions, the chronic care model, and different types of literacy in the award-winning series, *This is Us*.

**Results:**

Findings reveal that Randall’s experiences with mental health (*N* = 38 episodes) depict moments of narrative coherence and fidelity to varying degrees. We see Randall’s experiences align most with the self-management support and community elements of the CCM, but the overall depiction is unbalanced. Randall’s literacy level is high, but if inspected on a deeper level, analysis points to differing levels of health and mental health literacy, thus enabling and constraining positive and realistic portrayals of mental health.

**Discussion:**

Implications for mental health as a chronic issue and care delivery through CCM are discussed along with the importance of different types of literacy for audience members who may be struggling with a mental health disorder or trying to navigate the health care system. We offer recommendations for using Randall’s narrative as a teaching tool, integrating CCM into clinical visits to help guide delivery of care and understand literacy levels, and finally future work should continue this line of work from an Entertainment-Education perspective.

## 1. Introduction

Mental health is a global concern with high rates of depression and suicide (1, 2). Outcomes of unacknowledged and untreated severe mental health issues have negative outcomes such as developmental goals and premature death. Mental illnesses are common health conditions in the United States for people of all ages (3, 4). Anxiety is one of many mental health conditions that is characterized by feelings of worry or fear and includes several different forms generalized anxiety disorder, panic disorder, social anxiety disorder, separation anxiety disorder (5). Mental health treatments can be provided for relatively low cost, but the chasm between those who need help and those who actually seek and receive help remains deep (3, 5, 6). The uncertainty, fear, and lack of social connection because of lockdowns and high levels of stress related to COVID-19 only exacerbated the need for better mental health resources resulting in a shift in health care delivery services (7–11). Not only did COVID-19 increase mental health care needs and changed methods of health care delivery, but it also increased the amount of time people spent watching television (12–14).

One way people receive information about mental illnesses is through mainstream media, specifically television (15, 16). Research examining mental health depictions in media have typically been negative, involving characters who are stigmatized, painted as “dangerous” or “unpredictable,” and inaccurate, highlighting unusual or rare mental illnesses rather than those that are more mild and common (17–20). Going beyond the screen, the impact mental health portrayals have on audiences can carry over into mental health practice (21, 22). We argue that media representations of mental health, especially how care is delivered and received, is valuable for audiences and practitioners. We therefore bring in the Chronic Care Model (CCM) to guide analysis. Further, literacy levels, including reading levels and information comprehension about mental health issues is important in our understanding of why characters think or react to mental health information in a particular way (21, 23, 24). The current study uses narrative analysis to examine the narrative probability and fidelity at the intersection of mental health depictions, the CCM, and literacy in the award-winning series, *This is Us*, which has received praise for its depictions of mental health through the character Randall (25). Sterling K. Brown, the actor portraying Randall, also received many awards for his performance in *This is Us* (26), specifically related to his mental health narrative.

Previous work has focused on Randall’s mental health journey including access to mental health care and how seeing positive representations of a Black man receiving help can be beneficial for audiences and providers (22, 25). We build upon this work by including all six seasons using a qualitative narrative approach to analyze Randall’s experience navigating his chronic anxiety. Findings reveal a total of 38 episodes which relate specifically to Randall and his mental health journey. Analyzed episodes pertain to how Randall experiences, processes, manages, and treats his anxiety in line with the CCM and aspects of health literacy. Overall, Randall’s experiences align most with self-management support and community elements of the CCM, but the general distribution of factors are unbalanced. Randall’s literacy and educational levels are high, but if inspected on a deeper level, analysis points to differing levels of both health and mental health literacy, thus enabling and constraining positive, and realistic, portrayals of mental health. While *This is Us* presents a coherent narrative as an award-winning television show, Randall’s story illustrates varied levels of coherence, while narrative fidelity according to the CCM and literacy levels can be considered low. Implications for mental health as a chronic issue and care delivery through CCM are discussed along with the importance of different types of literacy for audience members who may be struggling with their mental health or trying to navigate the health care system. We offer recommendations for using Randall’s narrative as a teaching tool, integrating CCM into clinical visits to help guide delivery of care and understand literacy levels, and finally future work should continue this line of work from an Entertainment-Education perspective.

### 1.1 Media Depictions of Mental Health

For decades, we have seen media go beyond entertainment, impacting individuals in terms of their real-life attitudes, beliefs, and actions (19, 27–30). While this is true surrounding many topics, such as family planning (31), adult literacy (32), and sexual health (33), we also see that media have a significant impact in the field of mental health (16, 20, 34, 35, 36). Mediated depictions of mental health are both diverse and complex, ranging from accurate and sensitive depictions to stigmatizing and sensationalized portrayals. As shown in previous studies, we often make rapid judgments surrounding individuals with mental illness in media, whether real or fictional (34–37). Through understanding mental health depictions in the media, the importance of accurate depictions cannot be overemphasized. Because audience members have the potential to be influenced in a positive or negative way. This highlights the importance and responsibility of writers and producers to promote responsible, accurate and empathetic portrayals of mental health in their content (16, 34). These judgments effect real-world beliefs and attitudes and depend on whether a media portrayal is positive (38) or negative (39, 40).

When depicted in a positive manner, portrayals of mental health can help educate and raise awareness about mental health and treatment for audiences and clinicians (22, 30, 38). Portrayals of successfully treated mental illness can lead to less discrimination and social distancing among audience members (41). However, when portrayed as untreated, discrimination and social distancing increase (41). Positive portrayals can increase understanding and empathy among audiences, and in turn, facilitate positive societal change (42). For example, *Homeland* (2011) received a Voice Award to recognize its contribution in relation to education on mental health issues (38). Unfortunately, such positive portrayals are far and few between.

Many forms of entertainment media show characters with mental illness as violent or unpredictable, romanticize mental illness, or use mental illness as a humorous scapegoat (43). Such portrayals often reinforce negative stereotypes, support misinformation, and belittle health conditions and experiences (19). Negative portrayals have been found to influence individuals’ willingness to seek help from a psychologist, with heavy television viewers being less likely to participate in therapy (40). Popular primetime dramas such as *Criminal Minds* and *Law and Order* represent mental illness as violent and isolated, perpetuating this stigma and potentially leading to mentally ill viewers to self-stigmatize (44). Not surprisingly, individuals who receive most of their information on mental illnesses through television hold more negative and stigmatizing views in their real life (39). Diefenbach and West (39) found that heavy viewers of television negatively depicting mental illness were more hesitant to live near an individual with mental illness and did not support mental health services in their community. The popularization of these media depictions and their effects indicate a dire need for portrayals to be educational and informative, as well as entertaining (35). This is even more true for marginalized audiences with mental health issues as depictions of minority characters tend to uphold such negative stereotypes (22). While much of the work in mediated depictions has been on portrayals and stigma of mental health, less has been researched about the idea of delivery of care within a specific health care system. Thus, we look to the CCM to understand how chronic care is managed and portrayed within *This is Us*.

### 1.2 The Chronic Care Model

Understanding anxiety through the lens of chronic illness underscores the importance of providing patients with effective, patient-centered care that addresses symptoms and underlying factors which can inhibit quality of life across the lifespan (45–49). According to the National Institute for Mental Health, of the 14.1 million adults with a serious mental illness, approximately half of those individuals received mental health treatment (50). Specifically in the U.S., barriers such as affordability and environmental factors hinder successful treatment (51). Overtime, the CCM has been an effective tool in enhancing the treatment of a diverse set of chronic illnesses, including diabetes (52, 53), kidney disease (54), endometriosis (55) and various mental health issues (56). Overall, the CCM has become a widely adopted care approach, guiding health care initiatives at both community and national levels (57). We draw on the CCM to develop a deeper understanding of what better care might look like and what factors to facilitate care (58, 59).

The model identifies six components which contribute to successful chronic care health and management (60); *health systems, community, self-management support, delivery system design, decision support, clinical information systems*. First, the *health system* component refers to organizations, mechanisms, and the overarching culture that allows for safe, high-quality care. This includes positioning chronic care as a foundational goal of the organization, prioritizing it among both care providers and executives. Second, *community* relates to how social systems mobilize resources to meet patient needs. Encompassed in this is participation in and partnerships with community programs with the purpose of filling gaps in care. Advocating for policies which enhance patient care are also included here. Third, *self-management support* focuses on the role patients play in their own health and well-being. This aspect of the model focuses on how patients can be *activated patients* (61), meaning they are knowledgeable, equipped, and confident in managing their chronic illness. Fourth, *delivery system design* looks to assure effective, efficient care and self-management support. Here, this is achieved by implementing evidence-based care, clearly defining roles among care teams, continuing with follow-up care, and making sure patients clearly understand the medical plans prescribed to them. Fifth, *decision support* looks to promote care that is consistent with patient preferences and current scientific data. Finally, *clinical information systems*, explores how health systems utilize data to carry out efficient and effective care. Examples of this component includes providing timely reminders for all parties involved in one’s care plan and making sure information is share with the proper individuals to coordinate care.

The way in which patients can access care has changed in recent years. The COVID-19 pandemic led to an increase in patients receiving care and support through tele-medicine. Digital health interventions allow patients to access care using virtual means and include synchronous and asynchronous contact with a therapist, web-based peer support, mobile-based therapy programs, virtual and augmented reality, cognitive training, as well as technology that allows providers to monitor the ongoing health of the patient and adherence to prescription use (62). The implementation of these new technologies can offer flexibility for patients who are unable to receive in person medical care, but the lack of accessibility for specific vulnerable populations poses to be a challenge as well (11). Patients and providers may be faced with poor technological literacy and concerns such as inadequate IT structure to support the implementation of these new technologies as well as concerns related to privacy and safety. Although the COVID-19 pandemic helped to encourage the use of technology to deliver care, additional research is needed to further identify the most effective methods and identify and work through barriers that exist with digital health interventions related to mental health care.

The CCM is an appropriate model to examine mediated depictions of mental health because researchers can analyze not only how media content represents mental illness, but also how it depicts delivery of care and treatment. As we analyze Randall Pearson’s journey with anxiety, we harness the CCM to better understand his health experiences, to better evaluate how media portrays care for chronic conditions such as anxiety, and how this may impact viewers in their perceptions and own personal journeys navigating similar struggles. Thus, we ask:

RQ1: How do depictions of Randall’s mental health align with the CCM?

We argue that the CCM can also be used to enhance mental health literacy by promoting more accurate, realistic, and compassionate representations of mental health. What we mean by this is that if we can identify the gaps in Randall’s mental health care according to the CCM, then scholars and practitioners can be more focused on the message construction, meaning, and delivery of mental health information for viewers and patients as they consume entertainment media. Extending literacy beyond reading or writing messages related to mental health can reduce stigma and discrimination towards those working to manage and treat anxiety or other mental health conditions. Here, we look at how mental health literacy can be used by audiences to help make sense of mental health storylines in the media. We start with an overview of literacy in general and then delve into specific types of literacy.

### 1.3 Literacy

Literacy is an important staple of human development. The Organization for Economic Cooperation and Development (OECD) references the Program for the International Assessment of Adult Competencies (PIAAC) to define literacy as “the ability to understand, evaluate, use and engage with written texts to participate in society, to achieve one’s goals, and to develop one’s knowledge and potential” (63). PIAAC reports different levels of literacy at which an individual is able to comprehend increasingly complex texts of information. According to the National Center for Education Statistics (NCES), slightly more than half of U.S. adults have English literacy skills sufficient at completing tasks that require comparing and contrasting information, paraphrasing, or making low-level inferences (64). According to a different report, around 130 million people of ages 16–74 cannot read at the sixth-grade level (65). Given that a large proportion of Americans have low literacy levels, it is crucial we address this issue as they are costly financially. In a health context, low levels of literacy display higher hospitalization rates, poorer self-reported health, and higher costs of healthcare (65, 66).

According to the World Health Organization (67), health literacy can improve health and well-being across disparities. Personal Health Literacy, as defined by the Centers for Disease Control and Prevention (68), is the degree to which individuals have the ability to find, understand, and use information and services to inform health-related decisions and actions for themselves and others. An individual with high health literacy actively utilizes health information in their life, not just understands it. On the other hand, low health literacy may result in discrepancies between professional consensus on the most appropriate treatments for mental health care and the public perception of treatments (69). Stigma may play a role in low health literacy amongst caregivers as informal sources are preferred when seeking help regarding mental health support (70). Interventions that reduce stigma around mental health can help to increase ability to recognize a mental disorder and can increase instances of health seeking behavior and increase knowledge about the range of professionals available and what potential treatment options exist (71).

### 1.4 Mental health literacy

Mental health literacy, a distinct domain of health literacy, has also been receiving increasing attention from a global perspective (72). Mental health literacy encompasses many factors and includes knowledge around prevention of mental disorders, the ability to identify a developing mental disorder, knowledge of the proper channels to seek help, understanding self-help strategies, and how to support others afflicted with mental health problems (70, 73–75). Scholars have indicated that while some strides have been made, there is still a long way to go, and mental health literacy requires community members to come together to provide support for individuals with mental health disorders. Mental health literacy is critical as research indicates that many people do not know the psychological differences in specific disorders, may be avoidant toward seeking out information about disorders, may feel stigma around a disorder, and misinformation around disorders may be prevalent (69–71, 76). With the present issues concerning literacy, we examine how *This is Us* portrays mental health and whether such portrayals enable or constrain literacy toward its audience. Thus, the following question was posed:

RQ2: How do portrayals of Randall’s mental health enable or constrain different types of literacy?

## 2. Materials and methods

The current study is part of a larger project that examined health portrayals of the characters in the television show. We narrowed the scope of the study here to focus on Randall’s mental health journey across all six seasons and through the theoretical lens of both CCM and literacy. A qualitative narrative analysis was conducted to answer the proposed research questions.

### 2.1 Data collection

We accessed the series through Hulu streaming service. To compile the specific data set for this project, we began by reviewing the entire series codebook for portrayals of mental health. This included examples from Kevin, Kate, and Randall, all who struggled with various aspects of mental health, including weight, alcoholism, and diverse stressors. We then narrowed the scope again to focus on Randall specifically because of the popular press and academic work on depictions of his role in early seasons (22, 25). We compared our initial code book for content of mental health with summaries of each episode using online resources (e.g., Wikipedia/Vulture) to make sure Randall was the main character dealing with mental health concerns. In cases when the codebook and summary did not align, or there were insufficient details in the summary to make an informed decision to include or exclude an episode, we reviewed the episode and came to a consensus on the decision.

Next, we divided the episodes among teams of two to review and specifically code for depictions of Randall where he experienced extreme stress, anxiety, conversations about anxiety with family and community members. We acknowledge the difficulty in creating clear, defined, boundaries of what is and is not portrayed as mental health concerns. As such, we focused on the behaviors, key messages, and interaction among characters in Randall’s life. When there was disagreement among coders, it was brought to the group for discussion on inclusion or exclusion and we reached consensus. Once we had a complete list of episodes that portrayed Randall’s experiences with his mental health, *N =* 38, began our analysis. We created a codebook for the Chronic Care Model (58, 59).

### 2.2 Data analysis

To begin, we first argue that *This is Us*, is appropriate discourse for narrative analysis (77). Even though narrative conceptually, theoretically, and analytically has shifted and changed throughout the decades, we follow the definition wherein “a personal narrative is a distinct form of communication: It is meaning making through the shaping of experience; a way of understanding one’s own or others’ actions; of organizing events, objects, feelings, or thoughts in relation to each other; of connecting and seeing the consequences of actions, events, feelings, or thoughts over time (in the past, present, and/or future)” (78). What the overarching essence of narrative perspective allows is the ability to examine communication and experiences through stories and meaning making (77–81). Narrative probability or coherence refers to how the story hangs together and makes sense, while narrative fidelity is whether the story holds up to good reason. In this study, we evaluate Randall’s mental health experiences through the lens of CCM and literacy to identify these factors.

**Figure 1:**
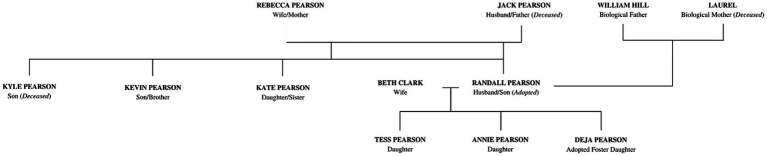
Character map of Randall’s family tree.

**Figure 2:**
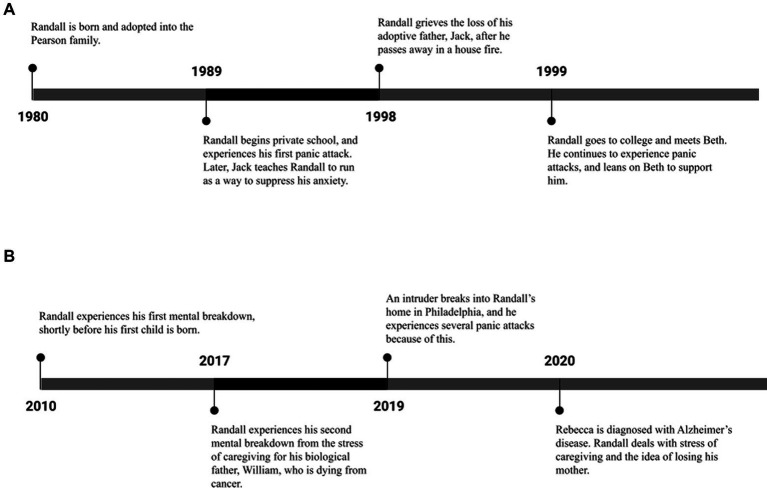
**(A)** Randall’s childhood mental health timeline and triggers. **(B)** Randall’s adult mental health timeline and triggers.

We review characters involved in the storyline, context, timing, and history of Randall’s character to help make sense of his mental health experiences. We grouped depictions chronologically based on Randall’s life, but not necessarily following the progression of the show as it utilized flashbacks and flashforwards. A character map of Randall can be seen in Figure 1, along with a timeline of his mental health journey and triggers throughout his childhood (Figure 2A) and adulthood (Figure 2B). Examples of narrative themes that compounded stressors and illustrated high anxiety throughout the show include navigating his identity in a transracial adoption, education-related anxiety, dealing with grief and loss of close family members, working as a member of the Philadelphia City Council, and balancing his family life as a husband and father of three children.

To evaluate narrative fidelity through RQ1, we constructed the codebook with definitions and possible examples from the series to guide analysis. We held team meetings to go through the codebook together and then assigned equal episodes among the team, two coders per set of episodes. This was done to ensure inter-coder reliability as each author watched the episodes independently and coded for the absence or presence of a specific piece of the CCM within each episode. The team also provided additional notes and summaries to further understand Randall’s story. When all coding was done, one person went through all the codes and marked any episode where there was a disagreement. Then the entire team came together and discussed the disagreements to decide by consensus for what the coding should be. Once we all had complete agreement, we were able to calculate the frequency of each element of the CCM for each episode, season, and entire series. We then plotted key narrative elements into the timeline for Randall’s mental health experiences and visually mapped out the portrayal of the CCM. The final step in our analysis to answer RQ2 was reviewing the depictions of his experience through the lens of literacy to understand how they enabled or constrained his own mental health literacy and the implications for viewers media literacy.

### 2.3 Ensuring Rigor

We want to highlight our efforts to ensure qualitative rigor and trustworthiness. To begin, the first author is PhD trained in qualitative methods and has over 10 years of experience with qualitative analysis. Remaining authors have different levels of educational and content training and bring with them unique perspectives related to biopsychosocial health experiences, thus enriching diverse analytic conversations. We integrated multiple qualitative inter-coder reliability checks. When there was disagreement that could not be resolved through discussion between the two coders, the conflict was brough to the whole team and we re-visited the content and engaged in consensus decision-making. We acknowledge that we are not in the same position as the character Randall, but we are able to remain grounded in the previous literature and focus on the communicative aspects how his story is portrayed.

## 3. Findings

We argue that Randall’s mental health was depicted (*N* = 38 episodes) as chronic as we follow him throughout his life (see Figure 1). Randall’s narrative demonstrates coherence in that we follow his journey in life as he manages his stressors in a variety of ways, which will be described in further detail below. The story hangs together as the depictions of life’s stresses and struggles make sense in a way that connects with the audience. Furthermore, based on the evidence that the show has won multiple awards, our argument is strengthened that the show and Randall’s portrayals of mental health present a coherent story.

In terms of narrative fidelity, due to the flashbacks and flashforwards, the chronology of Randall’s story could be difficult to follow and identify where in the storyline each mental health depiction is taking place. For example, we see Randall’s second breakdown first in Season 1 on screen, but only hear about his first that happened before audiences meet Randall and we never actually see it. Season four has the highest number of mental health portrayals (14), Season 2 and 6 have only one episode each that depicted Randall’s mental health, see Table 1. The incorporation of Covid-19 into the storyline in Season 5 would have seen Randall’s anxieties heighten even more due to the global pandemic. Even though there were many mental health depictions in Season five (*n* = 13), they were not related to Covid-19. With a general sense of narrative probability and fidelity in the show, we delve into the specific theoretical lens for narrative analysis, CCM, and literacy.

**Table 1:** Distribution of CCM factors per season.

	Season 1 *N = 3*		Season 2 *N = 1*		Season 3 *N = 2*		Season 4 *N = 18*		Season 5 *N = 13*		Season 6 *N = 1*	
	*n*	*%*	*n*	*%*	*n*	*%*	*n*	*%*	*n*	*%*	*n*	*%*
Health system *N* = 5	1	2.6	0	0	0	0	4	10.5	0	0	0	0
Community *N* = 12	1	2.6	1	2.6	1	2.6	4	10.5	3	7.9	1	2.6
Self-management support *N* = 14	1	2.6	0	0	1	2.6	7	18.4	5	13.2	0	0
Delivery system design *N* = 4	0	0	0	0	0	0	2	5.3	2	5.3	0	0
Decision support *N* = 2	0	0	0	0	0	0	1	2.6	1	2.6	0	0
Clinical information systems *N* = 2	0	0	0	0	0	0	0	0	2	5.3	0	0

### 3.1 CCM and mediated mental health

Our first research question examined how Randall’s experiences aligned with the CCM. For a full breakdown of each component of the CCM per episode and season, see Tables 1, 2. Throughout the series, we do see Randall leverage certain parts of the model (such as community and self-management) heavily while displaying low engagement in other areas. Towards the later seasons, we see a more complete and holistic adoption of the model to his own care after receiving encouragement from his family. This leads to not only better care for Randall, but improved outcomes and symptom management as well.

**Table 2:** Mental health episodes by CCM factor.

	Season 1 *N* = 3	Season 2 *N* = 1	Season 3 *N* = 2	Season 4 *N* = 18	Season 5 *N* = 13	Season 6 *N* = 1
Health system *N* = 5	Episode 14 *“I Call Marriage”*	–	–	Episode 5* *“Storybook Love”* Episode 11* *“A Hell of a Week: Part One”* Episode 15^+^ *“Clouds”* Episode 17 *“Strangers: Part Two”*	–	–
Community *N* = 12	Episode 15 *“Jack Pearson’s Son”*	Episode 4 *“Still There”*	Episode 17 *“R&B”*	Episode 4 *“Flip a Coin”* Episode 5* *“Storybook Love”* Episode 11* *“A Hell of a Week: Part One”* Episode 15* *“Clouds”*	Episode 1 *“Forty”* Episode 12** *“Both Things Can Be True”* Episode 13 *“Brotherly Love”*	Episode 1 *“The Challenger”*
Self-management support *N* = 14	Episode 15 *“Jack Pearson’s Son”*	–	Episode 2 *“A Philadelphia Story”*	Episode 3 *“Unhinged”* Episode 5* *“Storybook Love”* Episode 8 *“Sorry”* Episode 11* *“A Hell of a Week: Part One”* Episode 14 *“The Cabin”* Episode15* *“Clouds”* Episode 17 *“After the Fire”*	Episode 1 *“Forty”* Episode 3** *“Changes”* Episode 6 *“Birth Mother”* Episode 12** *“Both Things Can Be True”* Episode 13 *“Brotherly Love”*	–
Delivery system design *N* = 4	–	–	–	Episode 15* *“Clouds”* Episode 16 *“New York, New York, New York”*	Episode 1 *“Forty”* Episode 3** *“Changes”*	–
Decision support *N* = 2	–	–	–	Episode 17 *“After the Fire”*	Episode 3** *“Changes”*	–
Clinical information systems *N* = 2	–	–	–	–	Episode 1 *“Forty”* Episode 3** *“Changes”*	–

*Indicates episode recommended for teaching materials fostering conversations about mental health literacy. **Indicates episode recommended for teaching materials fostering conversations about the CCM. +Indicates episode recommended for teaching materials fostering conversations about mental health literacy and the CCM.

*Self-Management Support*. Self-management support is something we see Randall engage in across the entire series and was the most common piece of CCM portrayed in the series in (*n =* 14 episodes). One example where we see this happening is when Randall first meets his biological father, William, and discovers that he has late-stage stomach cancer (S1, E3). Randall goes on extensive runs, trying to push down the feelings of grief and anger he feels, and is seen running and researching treatment options for William rather than sleeping. Later in the series, Randall again goes on lengthy runs while trying to balance the stress of his councilman job, and after his family home is broken into (S4, E10). In both examples, his wife notices this and asks him if everything is okay, to which he simply brushes off, using his running as a cover for functioning well.

A second way of coping and managing his anxiety, Randall submerges himself in fixing other people’s problems, and we interpret this as an avoidance mechanism. This is consistent throughout his life, as we see Randall put off dealing with his own mental health issues by preoccupying himself with his family and problems they may be having. From helping his mom following his dad’s death, to helping William amidst his cancer diagnosis, and aiding the man that broke into their home, we see Randall try to take control of his circumstances by fixing others’ problems rather than resolving his own.

*Community*. Next, we see community remain a consistent role in Randall’s mental health care and treatment. Across the show, this was present in *n =* 11 episodes. In the first half of the series (S1-S3), we see Randall rely heavily on his family as a source of community, specifically his wife, Beth. Even as the two were dating in college, we watch as Randall spends many nights staying over in Beth’s dorm room where Beth comforts him through panic attacks (S4, E11). This continues into their adulthood and marriage, where Randall often confides in Beth, and she supports him through several, serious mental breakdowns and mental health crises. Outside of Beth, Randall relies heavily on his brother, Kevin. Kevin is often Randall’s first call when he feels he is out of control mentally. Kevin even misses big events, such as the opening night of his play, to physically support Randall during a bad mental health episode (S1, E18). Starting in the fourth season, we see this dynamic shift, as Beth expresses how she can no longer provide support for Randall, and he needs to seek out other forms of help. Throughout the series we also see Randall and Kevin’s relationship in conflict many times, therefore, the weight of Randall’s mental health become too heavy to handle on his own using his immediate support system and Beth encourages him to seek help, if not for himself, for her (S4, E15). We also see community as part of Randall’s storyline expand as he attends support groups in season 5. This is following the death of George Floyd, and we see Randall connect with other transracially adopted individuals which allows him to process many of his childhood experiences growing up (S5, E3).

*Health System*. Across the show, this narrative was present across *n =* 5 episodes. For this aspect of the model, we see Randall engage with health systems when he meets his first therapist. This occurs in seasons four. Randall first goes to see a therapist after a break-in occurs in his Philadelphia home (S4, E10). While Randall makes an appointment with a highly rated therapist, he struggles to admit his struggles and the large impact they have had on him throughout his life. He often pushes back at the therapist’s counsel, but amidst this she helps him process why he is really seeking help and what is realistic in his journey. Although Randall is resistant at first, we observe a generally positive experience, promoting both safe and high-quality care.

*Delivery System Design*. Delivery system design was seen in *n =* 4 episodes as Randall’s first therapist, Dr. Leigh, strives to help him construct effective and meaningful self-management support strategies. This includes addressing some of his current strategies, including running and helping behaviors. As part of this, they work together to establish why Randall has sought out therapy and what his goals should be during this time. Here, Randall is also faced with how he is currently using his self-management techniques, how their role in his mental health is more harmful than helpful, and considered how he could use them moving forward in a healthier and more beneficial way.

*Decision Support*. Decision support was present in *n =* 2 episodes, while seeking professional care. The first time we see this occur is during Randall’s meeting with Dr. Leigh. During their conversation, she asks Randall why he came to see her, and discusses that according to research she was surprised Randall chose to go to her office. When Randall responds that she is a highly rated therapist in his area, she explains how there are other qualities to consider when choosing a therapist. Later in Season five, we see Randall take this into consideration when he decides to go back to therapy, selecting a therapist more in-tune with the areas he is struggling with, such as his race and identity. This decision proves to be beneficial to Randall, as he begins to analyze his own experiences, and the complexity of feelings such as isolation, identity confusion, and a sense of not belonging. These scenes emphasize the importance of culturally sensitive therapy, and as depicted, Dr. Vance is trained and equipped to address Randall’s unique experience and background.

*Clinical Information Systems*. Finally, clinical information systems were present in *n =* 2 episodes, seen only in Season five. As this season took place during the Covid-19 pandemic, we see Randall engage in therapy over Zoom with Dr. Vance and utilize technology amidst his care. During their meetings, they as navigate not only Randall’s anxiety, but also his experiences growing up as a transracially adopted child. This reflected an adjustment that not just the show, but a large majority of patients had to adjust to amidst the Covid-19 pandemic, as meeting in person was not a feasible option. Randall’s transition from in-person to virtual care is rather seamless, not reflecting any technological delays or struggles in navigating his scheduled appointment.

### 3.2 Literacy and mental health

Our second research question asked how Randall’s mental health narrative enables or constrains different types of literacy. Despite being a successful, highly educated, and publicly esteemed person, Randall demonstrated high and low levels of different types of literacy. In terms of overall literacy, Randall is highly intelligent. He attends a different private school than his siblings, he is on the path to Harvard, but chooses to pursue Howard University, and then eventually attends Carnegie Mellon after Jack’s death. Thus, even amidst carrying grief and guilt with him, he is able successfully finish college, acquire a high-level job in his field, and throughout his entire narrative, continues to grow in knowledge and achieve his fullest potential because of his ability to engage with texts, set goals, and accomplish them both professionally and personally. In fact, his traits of extreme focus and dedication to a goal, set him up to be very successful, including implications that he may 1 day run for President of the United States of America. We argue that Randall’s experiences with mental health do not interfere with his literacy, rather enables him to achieve at the highest level.

In terms of health literacy, Randall does many things to engage in a healthy lifestyle, relating to diet and exercise, and we argue he is well aware of the importance of taking care of his own biomedical health. We also see him enabling health literacy for others more than himself. For example, there are two times when his parents, non-biological mother and biological father, get diagnosed with terminal illnesses. Randall does everything within his power to search out treatments, make doctor’s appointments, and utilizing his strength in high literacy to make sure they receive the highest level of health care possible. What is interesting, is we see that this comes at a high cost to his own personal mental health as he does not put the same effort into acknowledging when his anxiety levels spike *and* seek help. At an individual level, he is constraining his own mental health literacy.

Randall’s journey with mental health literacy is a complicated one, influenced by both his adopted and birth parents. While Jack is the one who taught Randall how to exercise and control his breathing to manage his anxiety, he is also the one who dismissed his anxiety as a non-serious health issue. That said, in Randall’s childhood, Jack and Rebecca were not equipped themselves to understand Randall’s anxiety nor the proper channels to seek assistance with it.

Findings illustrate that until Season five, Randall holds a limited understanding and a reluctant view towards searching for effective treatment options (e.g., therapy). Previous to that, Randall’s experiences constrain mental health literacy. Although he is aware he has anxiety, he fails to identify his own triggers, signs of deteriorating mental health, and lacks the necessary skills to manage his mental health during high-stress situations. Such warning signs include (S1, E14): shaking hands, tremors, vision problems, an increased need for exercise, inconsistent sleep routines, and vivid dreams about his stressors and these lead viewers to see Randall’s first on-screen breakdown (S1, E18). After his daughter Tess has a panic attack (S4, E5), Randall tells her that he can relate and often does not know he is feeling anxious until it reaches the level of a breakdown. This is the first time Randall acknowledges that he does not know how to identify the symptoms of his anxiety until it is too late. After the home break-in, Randall attempts to exercise to relieve his anxiety but we see it bottle inside until he violently releases his energy on a burglar he encounters on the street. After this, Randall finally calls Kevin to confide in and seek help (S4, E11).

In his therapy sessions, we see Randall painfully process and work through the roots of his anxiety, but we do not see him focus on coping strategies. It was Randall’s original belief that he began therapy to work on his control issues, however, when the topic of Rebecca’s health is brought up, it is apparent that he holds resenting feelings toward her. Season five is where we observe Randall to have improved mental health literacy. He understands the proper channels to seek help for his anxiety, including his therapist, Dr. Vance, and his transracial adoptee support group.

## 4. Discussion

Findings from the current study of the intersection between mental health depictions, CCM, and literacy illustrate a coherent narrative, yet are lacking fidelity. *This is Us* presented audiences with a major storyline that depicted a highly successful Black, transracial-adopted male, growing up in a predominantly White community. From a very early age, we see depictions of Randall living with a mental health disorder and how that informs his story across six seasons of the show. Reinforcing previous research, we argue that using television to bring normally stigmatized topics to the forefront of audiences has the potential to affect viewers in a positive way (22, 25, 42). For the millions of viewers following the series, the writers provide a character who struggles to be everything for everyone, at the cost of his own mental health. What we noted of great importance from this analysis is that mental health care is hard. We often think that we can handle it on our own, but destigmatizing mental health care delivery is critical. We argue that is why Randall’s narrative journey from avoidance to accepting professional mental health counseling through therapy is valuable to audiences. Mental health does not have to be crippling and/or stigmatizing. Those living with mental health disorders can still be successful, both personally and professionally, and more realistic portrayals of how mental health care services are delivered could be even more beneficial.

### 4.1 Implications

*This Is Us* has been shown to facilitate meaningful conversations around complex issues (e.g., Alzheimer’s disease, caregiving, end-of-life) we see this also happening in relation to anxiety and mental health (22, 82, 83). Shows with positive depictions can serve as a starting point for conversations or an encouraging “push” to seek out care or help if needed. The entertainment appeal of these shows may help reach other populations as well. Even though one thinks they are doing fine on their own, the show breaks down the stigma surrounding going to a counselor. Also helpful was the narrative’s portrayal of therapy, specifically how going to therapy once did not solve all of Randall’s problems. Additionally, audiences watch Randall work through his experiences and emotions, which was not easy. This process also took time, as Randall worked with multiple therapists to find the one that fit his needs best and continued going to sessions.

### 4.2 Implications for CCM

As the CCM proposes, there are many factors which contribute to quality care for patients with chronic anxiety. While several factors are listed, it is important to note how all factors are equally weighted in their importance. Meaning, one can be missing certain elements in their health care experiences and still receive good care, but best care will involve all elements of the model. This is a critical point where we see the value of different types of literacy working together. In Randall’s case, for the first four seasons of the show, we see a strong imbalance in the factors outlined by the CCM and he works through many mental health challenges. What we learn from analysis is that quality care for mental health is a complex system and sometimes best advocates are those around you.

While self-management support and community are salient factors important to Randall’s narrative, and temporarily managed his anxiety, they prove to be an unsustainable way for him to manage his care. Through Beth, a character with higher mental health literacy who is a community pillar of support for Randall, he finally incorporates a more balanced approach to his care. Randall begins incorporating other factors of the model, resulting in more effective management of his anxiety. At the same time, as the more balanced his approach to mental health care is according to the CCM, we see improvements in his overall literacy, thus depicting a much healthier Randall. Even though Randall’s anxiety did not go away, it was more manageable and he was better able to care for himself and those around him. This was a learning process for Randall, one we watched him embark on throughout his life. For viewers, this is important because they see the longevity of Randall’s anxiety. Also, there is no perfect treatment or solution. Rather, overtime and through trial and error, we see him become gradually better as he finds balance in his treatment which is an important step in his care.

As many health campaigns have worked to overcome the stigma surrounding mental health, we argue that more public health efforts can be made to highlight community support, whether that be family, health professionals, support groups, friends, religious organizations, and so on, having someone to be able to talk to and share experiences, including mediated conversation starters (1). While Randall is literate, his mental health literacy is rather low, until he successfully connects with the second therapist and attends support group. Media health literacy did play a role in Randall’s narrative, but we also recognize that this is an area to delve into further for the audiences as they watch various television programs. Based on our findings, characters need a combination of all the different types of literacy to be fully successful in searching for and evaluating health information. Additionally, when we bring in the CCM, knowing what and when an area of care coordination is missing can be an incredibly valuable tool for both individuals and clinicians.

As we can see from our analyses, Randall receives exceptional care and has all resources accessible to him. On the positive side, we can see how this accessibility can and does work for him. He has the means to meet and work with several different therapists until he finds one that fits with his needs. He also has exceptional family support and he ends up incorporating several healthy coping mechanisms in his day-to-day life. On the negative side, one may not perceive this as realistic. The reality of accessibility is a huge factor, especially during and post pandemic times. What is really interesting is that *This is U*s incorporated COVID-19 into the storyline for characters, and is when Randall starts to seek out mental health care. This displays narrative fidelity in a unique way as we saw a shift in real life and in the series to tele-health appointments. Randall was able to manage his mental health successfully as the show continued but we argue that in reality, the changes in health care delivery of mental health care services continue to be in flux. Furthermore, a whole new level of literacy is introduced with issues of the digital divide, digital health literacy, and technology/connectivity issues that individuals and practitioners may not have control over. We recommend that more focus on delivery care systems can be helpful (1, 8, 84). At the same time, as mentioned above, none of Randall’s stressors during the pandemic were about covid, which could be seen as unrealistic.

The average person may not be able to be able to receive the same type of care that Randall has, thus setting unrealistic expectations for those who may be identifying with Randall. There are many things to consider regarding Randall’s care path. This includes insurance, doctor referrals, making appointments, time, and financial burdens; all of which never appear within the show. Moreso, we note that Randall never seeks help by himself for himself. At all points where Randall’s mental health was discussed, it was always a community effort to get him there. We can see this with Beth pushing him to see a counselor and joining a support group, all of which he never would have never approached on his own. Randall’s mental health journey has been a lifelong experience for him. We cannot understate how important the community and social support elements are in we hope to encourage successful mental health care delivery.

### 4.3 Practical implications for health practitioners

Given the popularity of *This is Us* coupled with the fact that many turn to media to learn about health issues, the present study also holds several practical implications for health care providers, specifically those training for or currently practicing in the field of psychiatry. Our analysis can help inform mental health practitioners and psychiatrists about the portrayal of mental health issues in popular media. We see this as a point of connection between patient and care provider (22). We offer three recommendations for mental health care professionals and scholars to move this line of research forward in providing care and future research.

First, we recommend using Randall’s experiences as a springboard for discussion starter in the high school or college classroom. While Randall’s entire narrative arc can be helpful for this, we also recommend specific episodes for breaking down stigma through discussion of Randall’s mediated experience with mental health, please see Table 2, where the suggestions are marked with an asterisk. For CCM, we suggest integration into more health science related materials, or the construction of continuing education modules focused on case study scenarios. We suggest episodes where audiences watch Randall receive mental health in the form of a therapist and a support group. For classes focusing more on health, mental health, or media, these same episodes would also be suggested as they depict conversations centered around various types of literacy. By using popular media content in the classroom targeted discussions surrounding breaking down the stigma with representations of mental health in the media can be beneficial.

The second recommendation focuses on the need to integrate the CCM as technique into clinical practice. We suggest presenting patients with an empty model with the components of the CCM and asking the patient to fill it out according to their experiences. The reflective nature of such an exercise could help practitioner and patient see the gaps in care and components of literacy in relation to understand mental health care and delivery. The mental health literacy of patients should be considered when planning care. It is important that patient education is addressed by both primary care doctors and more specialized care providers, such as psychiatrists. Anxiety disorders are among the most common mental health problems seen in primary care settings and general medical physicians can facilitate or impede access to more specialized care through their referrals (85). Understanding that a patient may find the most success with a provider who they feel is similar to them is important, especially when considering the finding that members of minority groups are often less likely to receive mental health treatment (85).

The possibility of both undiagnosed and diagnosed generational mental health conditions is important for providers to consider. Patients may be unaware of symptoms that they are displaying, leaving them unable to identify the initial symptoms before they lead to a more serious mental health emergency. Ignoring these symptoms may negatively affect their relationships with friends and family. When navigating through the process of managing anxiety, it is important to consider the role of community within each patient’s care plan. Working with their community and close support system they may find more success. Finally, identifying and managing healthy self-management techniques may also lead to more success for patients. Although Randall found running to help initially, it became a way of avoiding his anxiety rather than working through it in a constructive way. The portrayal seen on *This Is Us* might help to inform both doctors and potential patients who view the show and can relate to the portrayal of Randall’s anxiety journey.

The third recommendation is for continued work in this area to specifically use an Entertainment-Education perspective (86). Through interdisciplinary partnerships between physicians, scholars and writers/creators, such collaborations can help ensure positive and realistic portrayals of mental health and treatment options in popular media. Writers and content creators can harness creative skills to develop compelling stories and relatable characters that engage audiences to convey important health information. Working with physicians and scholars can offer clinical and theoretical insights to ensure that content is accurate and that it reflects current research recommendations and best practices. Together, these groups can create engaging content that helps educate and entertain about mental health, chronic care, and ultimately, improve mental health literacy. In turn, combined efforts may create more engaging storylines that can positively impact audience attitudes and behaviors when it comes to their own mental health or the mental health of a loved one.

### 4.4 Limitations and future directions

The present study is not without its limitations. First, while we are able to code and analyze portrayals of mental health in *This is Us*, we are only able to make comments on how mental health is depicted and cannot comment on the direct impact on audience attitudes and beliefs towards mental health and seeking out care. Future studies could implement an experiment to test these effects. Second, we recognize that manifestations of anxiety and mental health look different for everyone, and therefore, feasible and effective treatment may also look different for different people. Although Randall’s life experiences may not be generalizable for all audience members, we argue it can be used as a starting point for those interested in seeking care and treatment, and its incorporation into popular media can help destigmatize mental health.

Finally, while the present study evaluates Randall’s mental health journey with anxiety through the lens of the CCM and three types of literacy there are more related avenues to push this research forward. Another important aspect of chronic care which is important to consider is care coordination. As outlined in the care coordination model (87), effective communication also includes factors such as accountability, patient support, relationships and agreements, and connectivity. An important consideration here is the interactions between health care systems which can heavily impact patient outcomes. A major part of Randall’s storyline shows him receiving care, and while receiving care is important, viewers should also see what the process of getting that care looks like (including insurance, processing, and setting up appointments). We argue this is an equally important and realistic aspect of care and would help audiences better understand what this process might look like and where they could start. In terms of literacy, media literacy is critical for viewers as well and future studies should examine this (84).

## Data availability statement

The original contributions presented in the study are included in the article/supplementary material, further inquiries can be directed to the corresponding author.

## Author contributions

KT, TL, KH, EL, and MN were involved in the study conceptualization, planning, and design. KT, TL, KH, EL, MN, and SG contributed to data collection, creation of code books, episode coding, analysis and writing of the manuscript. All authors contributed to the article and approved the submitted version.

## Conflict of interest

The authors declare that the research was conducted in the absence of any commercial or financial relationships that could be construed as a potential conflict of interest.

## Publisher’s note

All claims expressed in this article are solely those of the authors and do not necessarily represent those of their affiliated organizations, or those of the publisher, the editors and the reviewers. Any product that may be evaluated in this article, or claim that may be made by its manufacturer, is not guaranteed or endorsed by the publisher.
